# Synchronized personalized music audio-playlists to improve adherence to physical activity among patients participating in a structured exercise program: a proof-of-principle feasibility study

**DOI:** 10.1186/s40798-015-0017-9

**Published:** 2015-05-08

**Authors:** David A Alter, Mary O’Sullivan, Paul I Oh, Donald A Redelmeier, Susan Marzolini, Richard Liu, Mary Forhan, Michael Silver, Jack M Goodman, Lee R Bartel

**Affiliations:** 1 Institute for Clinical Evaluative Sciences, 2075 Bayview Avenue, Toronto, ON M4N 3M5 Canada; 2 University Health Network Cardiovascular Prevention and Rehabilitation Program, Toronto Rehabilitation Institute-University Health Network, 550 University Avenue, Toronto, Canada; 3 Sunnybrook Health Sciences, 2075 Bayview Avenue, Toronto, Canada; 4 Department of Medicine, University of Toronto, 27 King’s College Circle, Toronto, Canada; 5 Department of Exercise Sciences, Faculty of Physical Education and Health, University of Toronto, 27 King’s College Circle, Toronto, Canada; 6 Music and Health Research Collaboratory, Faculty of Music, University of Toronto, 80 Queens Park, Toronto, Canada; 7 Dean’s Office, Faculty of Music, University of Toronto, 80 Queens Park, Toronto, Canada; 8 Department of Health Policy, Management and Evaluation, University of Toronto, 40 St George Street, Toronto, Canada; 9 Faculty of Rehabilitation Medicine, The University of Alberta, 8205 114 Street, Alberta, Canada; 10 The University of Toronto, 27 King’s College Circle, Toronto, Canada

## Abstract

**Background:**

Preference-based tempo-pace synchronized music has been shown to reduce perceived physical activity exertion and improve exercise performance. The extent to which such strategies can improve adherence to physical activity remains unknown. The objective of the study is to explore the feasibility and efficacy of tempo-pace synchronized preference-based music audio-playlists on adherence to physical activity among cardiovascular disease patients participating in a cardiac rehabilitation.

**Methods:**

Thirty-four cardiac rehabilitation patients were randomly allocated to one of two strategies: (1) no music usual-care control and (2) tempo-pace synchronized audio-devices with personalized music playlists + usual-care. All songs uploaded onto audio-playlist devices took into account patient personal music genre and artist preferences. However, actual song selection was restricted to music whose tempos approximated patients’ prescribed exercise walking/running pace (steps per minute) to achieve tempo-pace synchrony. Patients allocated to audio-music playlists underwent further randomization in which half of the patients received songs that were sonically enhanced with rhythmic auditory stimulation (RAS) to accentuate tempo-pace synchrony, whereas the other half did not. RAS was achieved through blinded rhythmic sonic-enhancements undertaken manually to songs within individuals’ music playlists. The primary outcome consisted of the weekly volume of physical activity undertaken over 3 months as determined by tri-axial accelerometers. Statistical methods employed an intention to treat and repeated-measures design.

**Results:**

Patients randomized to personalized audio-playlists with tempo-pace synchrony achieved higher weekly volumes of physical activity than did their non-music usual-care comparators (475.6 min vs. 370.2 min, *P* < 0.001). Improvements in weekly physical activity volumes among audio-playlist recipients were driven by those randomized to the RAS group which attained weekly exercise volumes that were nearly twofold greater than either of the two other groups (average weekly minutes of physical activity of 631.3 min vs. 320 min vs. 370.2 min, personalized audio-playlists with RAS vs. personalized audio-playlists without RAS vs. non-music usual-care controls, respectively, *P* < 0.001). Patients randomized to music with RAS utilized their audio-playlist devices more frequently than did non-RAS music counterparts (*P* < 0.001).

**Conclusions:**

The use of tempo-pace synchronized preference-based audio-playlists was feasibly implemented into a structured exercise program and efficacious in improving adherence to physical activity beyond the evidence-based non-music usual standard of care. Larger clinical trials are required to validate these findings.

**Trial registration:**

ClinicalTrials.gov ID (NCT01752595)

**Electronic supplementary material:**

The online version of this article (doi:10.1186/s40798-015-0017-9) contains supplementary material, which is available to authorized users.

## Key points

- The integration of tempo-pace synchronized preference-based music audio-playlists into structured exercise program resulted in significantly greater weekly physical activity volumes than did non-music usual-care.
- Differences in weekly physical activity volumes were greatest among those receiving rhythmic auditory stimuli embedded into their audio-music playlists.
- A group’s average weekly physical activity positively correlated with their use of audio-playlist devices.

## Background

Physical activity has irrefutable benefits to quality of life and survival [1,2]. However, suboptimal uptake and adherence undermines the survival and health benefits associated physical activity in real-world settings [3]. Among patients with established cardiovascular disease, structured exercise programs have been used to promote the self-management and adherence of physical activity, [4] but their effectiveness has also been undermined by high rates of behavioural attrition to physical activity [5,6]. Strategies to improve adherence to physical activity among such populations have proven challenging often yielding inconsistent results [6,7].

Music rhythmicity has been shown to modulate the perceptions and ergonomics of physical activity [8-16]. Postulated mechanisms of action have included neural-entrainment effects and movement cueing step-cadence regulation [8,17-30]. When coupled with individual preferences, such music may further enhance motivation, improve affect, induce distraction and attenuate patients’ perceptions of effort during exercise [19,21,26,27,31-33]. While integrated mobile music audio-devices are widely available, no study has explored the feasibility and efficacy of using synchronized preference-based music audio-playlists to improve adherence to physical activity.

The objective of the Music Activity INTervention for Adherence Improvement through Neurological entrainment (MAINTAIN) study was to explore the feasibility and efficacy of implementing tempo-pace synchronized personalized audio-playlist devices to improve adherence to physical activity among patients participating in a structured exercise program. All patients received usual care (cardiac rehabilitation). Patients were randomized to either usual-care alone (i.e. no-music) or an integrated music strategy consisting of personalized customizable audio playlists uploaded onto iPods® whose song selections were comprised of tempos (beats per minute) which matched the individuals’ pre-specified walking/running pace as prescribed by the structured exercise program itself. As part of our proof-of-concept feasibility study, among patients randomized to the integrated music strategy, we evaluated the incremental feasibility and efficacy of embedding additional rhythmic sonic enhancements to the customized audio-playlists (herein termed rhythmic auditory stimulation (RAS)) to explore whether beat-accentuation could further improve exercise adherent behaviours over and above non RAS synchronous music [29].

## Methods

### Research setting

Cardiac rehabilitation served as our test-case setting for structured exercise for several reasons: First, it is an established evidence-based guideline-driven structured exercise and lifestyle modification program which has been shown to improve survival in cardiovascular disease [34] and therefore represents an ideal minimum ‘gold-standard’ level of care for all patients irrespective of treatment allocation. Second, exercise prescriptions, based in part on initial cardiopulmonary assessments, guide an individual’s exercise pace. Third, as with other structured exercise programs, cardiac rehabilitation is associated with significant behavioural attrition, which are characterized by high-rates of programmatic drop-out and poor adherence to the self-management of physical activity [35]. Accordingly, a music tempo-pace synchronization strategy theoretically lends itself to the cardiac rehabilitation environment because it can help regulate, maintain and reinforce the exercise pace as prescribed by the program itself.

All study patients had established cardiac disease and were actively participating in the Cardiovascular Prevention and Rehabilitation Program at the Toronto Rehabilitation Institute-University Health Network (TRI-UHN) [35]. Patients were originally referred into the program by physicians or through automated referrals following cardiac hospitalization. All patients received cardiopulmonary and anthropometric baseline assessments and attended on-site exercise classes once per week. An assigned case-manager prescribed aerobic and resistance exercise activities to be conducted at home on at least four additional days per week. Each patient who attended the program was followed for 6 months, and re-evaluated at month 3 and month 6.

### Sample eligibility criteria

We included English-speaking patients with known cardiovascular disease participating in cardiac rehabilitation. Exclusion criteria consisted of patients with a history of head trauma/seizures (a relative contraindication to RAS) [36] and those unable to wear headphones due to hearing impairment or hearing aids to ensure that patients had sufficient exposure to the music intervention. Patients were recruited during the third-class of the program (corresponding to a pre-screening period of 3 weeks from program onset).

Study enrollment was restricted by funding. However, we projected that such resource restrictions would still allow for sufficient enrollment to yield a minimum effective sample size that would be sufficient for primary outcome analyses, even after factoring in an anticipated drop-out rate of 10% per group (see sample size justification below) [4,5,37].

The recruitment process began in a group setting, consisting of a cardiac rehabilitation class comprised of between 20 and 25 patients. Patients were informed about the trial within a 5 min presentation following patients’ third pre-scheduled cardiac rehabilitation class. In total, 139 patients were screened for the study. Enrollment was confined to the first 34 patients who volunteered and met inclusion/exclusion criteria (all 34 patients who volunteered met eligibility criteria). There were no significant differences in the age and gender of study sample as compared with the non-consenting cardiac rehabilitation classes from which they had been recruited (Additional file 1).

### Study design

The study was primarily designed as a proof-of-concept feasibility study to explore the implementation and efficacy of integrating personalized music preferences and tempo-pace synchronization into a structured exercise program.

We incorporated a randomized experimental design. The randomization process incorporated a two-step process in which patients were first randomized to music audio-playlists vs. no music audio-playlists (i.e. 2:1 ratio of music vs. no-music audio-playlist, respectively). Among those randomly allocated to music playlists, patients were further randomized (in 1:1 fashion) to RAS vs. no RAS groups. All patients irrespective of music received usual-care (cardiac rehabilitation). The incorporation of a two-step rather than one-step randomization process allowed us to first explore our primary hypothesis: namely, personalized audio-playlists with tempo-pace synchronization would result in improved adherence to physical activity as compared with a gold-standard non-music usual-care control. The second step in the randomization process allowed us to more specifically explore the implementation of RAS (vs. no RAS) among those initially randomized to music audio-playlists, and do so in blinded fashion.

All randomization incorporated the use of a random-number generator; patient names and study numbers were kept within sealed envelopes during the randomization process to preserve blinding of the randomized-group allocation process.

MAINTAIN was designed to examine the incremental effects of music playlists on the usual-care programmatic delivery of cardiac rehabilitation administered by case managers irrespective of MAINTAIN’s treatment allocation. Case managers involved in the administration of cardiac rehabilitation were blinded to all metrics related to the study. In addition to the minimum standard of cardiac rehabilitation care, all three randomized groups received tri-axial accelerometer activity monitors (PAM® Personal Activity Monitor, Model AM300, Pam, Doorwerth, The Netherlands) and self-reported physical activity diaries for use throughout the entire study duration. Each individual irrespective of randomization interacted with the study coordinator every 2 weeks for a 3- to 5-min duration to collect and upload activity diaries and information obtained through the audio-playlist device. During these encounters, patients randomized to music (with or without RAS) had the opportunity to request additional playlists or modifications to playlists that reflected changes in an individual’s prescribed exercise pace (if appropriate); such changes were then uploaded on the subsequent scheduled encounter.

### Music playlists, tempo-pace synchronization and RAS implementation

Patients randomized to personalized music playlists also received a personalized audio playlist device (iPod® mp3 players), which were preloaded with a playlist of music that satisfied their pre-specified music genre and artist preferences. Music preferences were ascertained at the outset of the study. Patients were asked for examples of music genres (e.g. styles such as rock, jazz, pop, classical, country/western, folk), artists, albums and songs they preferred to listen to, and preferred not to listen to, while exercising.

All music assembled within playlists was synchronized to approximate an individual’s prescribed exercise pace. Specifically, commercially available songs selected for music playlists were chosen such that tempos were within ±10 beats per minute of a subject’s exercise step-pace as specified by the initial exercise prescription. The incorporation of a maximum 10 beat per minute pace-tempo threshold was based on pre-trial pilot data and qualitative feedback on motivation and perceived exertion with music during exercise. Moreover, evidence suggests that a 10 beat per minute or less tempo-pace calibration difference falls within thresholds by which patients can feasibly adjust their walking/running pace to accommodate to sonic and music rhythms by subconsciously increasing or decreasing their strides accordingly [18]. Exercise pace was ascertained by determining the number of steps taken within a quarter mile in a time duration as ascertained by the cardiac rehabilitation program. The number of steps taken per minute at a target pace was used to generate the optimal tempo of music required for tempo-pace synchronization. Each quarter note was intended to correspond with one paced step. We created a database comprising over 14,500 songs of various genre, artist, title, song duration and music tempo. The music tempo of each song was determined using commercially available software (cadence® software) [38].

The process of RAS enhancement adapted the methodology used by Chen et al. in which each quarter note beat was enhanced through the addition of an audible rhythmic sound [39]. RAS enhancements were conducted in a random subset of songs within an individual’s playlist (comprising up to 50% of an individual’s personalized playlist). Patients were not told which songs were RAS-enhanced, or which of the two music groups (RAS vs. no RAS) they were allocated to. Our rationale for leaving some of the songs unmodified was to help us ascertain, through focus-group interviews (see below), whether patients could distinguish between RAS and non-RAS songs uploaded into their individual playlist. Pre-study pilot testing further informed the frequencies and volumes used for RAS enhancement. Our intent was to implement RAS enhancement at frequencies and volumes just beyond minimal detection levels without detracting from the authenticity of the music playlists. The RAS-enhancement process first imported the original mp3 playlist audio files into Logic ® 9.0 recording software. RAS-enhancements were conducted using sounds of drums (kick-drum, snares, hihats and rides) through the addition of secondary tracks and digital keyboard MIDI-drum instruments, played concurrently with the original playlist file. Each song was then ‘sonically modified’ manually to accentuate and enhance the quarter-notes through the use of lower and/or higher frequency drum instruments. Lower frequency drum sounds (e.g. kick-drum at 50 to 100 Hz) were generally maintained at volumes of between 1 and 5 dB louder than the original recording, whereas higher frequency drum sounds (e.g. snare or hihat at 1,000 to 5,000 Hz) were generally maintained at volumes of between 1 and 5 dB lower than the reference original music track. RAS-enhanced mp3 songs were then re-mixed and mastered as a WAV file (44 kbit/24 bit) and subsequently compressed into an mp3 file and uploaded into the RAS iPods® playlist so that maximum ‘headphone’ volumes were similar to the original audio-recordings. In short, RAS enhancements accentuated the frequencies of externally embedded rhythmic components relative to the other frequencies and sounds of the original audio-file, without altering the total volume of the sound recording itself (Additional file 2).

The sonic attributes of those randomized to RAS enhancement resulted in significantly higher peak frequencies, more abrupt slopes of peak frequencies, selected higher peak volume frequencies relative to baseline and lower frequency levels at peak frequencies (Additional file 3).

Data on baseline variables included age, gender, body mass index (measured weight and height), functional capacity, self-reported depression-score, self-efficacy, referral indication, cardiovascular risk factors and prior disease. Depressive symptoms were ascertained using the Centre for Epidemiological Studies - Depression Scale (CES-D), while exercise self-efficacy was ascertained using the Stanford Self-Efficacy scale; both measures have been validated [40,41]. In addition to data collected through empirical methods, information about the perceived impact (if any) that music had on patient participation in their prescribed exercise program was collected through the use of small group interviews in which participants were asked to describe their exercise experience. Specifically, participants were asked for their opinion about the impact of listening to music on their exercise behaviour. Participants were interviewed in small group settings within their respective treatment allocation arms.

The study was reviewed and approved by the Toronto Rehabilitation Institute-University Health Network Research Ethics Board. All procedures followed were in accordance with the Helsinki Declaration of 1975, as revised in 2008 (5).

### Outcomes

The primary outcome was defined as the weekly average volume of physical activity (i.e. mean weekly minutes of physical activity as measured using tri-axial accelerometers) throughout the 3-month study. Sample size estimates incorporated the methods by Hedeker for repeated measures (weekly activity levels) and assumed a correlation of 0.4 in activity levels over 12 weekly interventions [42]. The minimum effective sample size targeted for this study was 10 patients in each of the three groups, which would allow for 80% power to detect a clinically large size mean differences (i.e. 50% or 250 min per week of physical activity) between the music and no music treatment groups, assuming a two-tailed alpha of 0.05. The sample size was only powered sufficiently to account for group-level differences using a repeated-measures design but underpowered to account for variations within and between individuals. Secondary outcome measures consisted of the weekly volume of intensity-specific physical activity as defined as high-intensity, (≥7 metabolic equivalents), moderate intensity (3 to 6.9 metabolic equivalents) or low intensity (<3 metabolic equivalents), based on the PAM® personal activity monitor. Validation of the PAM® accelerometer has been published elsewhere [43].

### Statistical analyses

Given the staged sequential randomization process, statistical analyses first compared usual-care with the combined music intervention groups (with RAS and no RAS combined). Only if significant differences in weekly physical activity volumes between music and non-music groups existed, did we examine differences in outcomes across the three intervention groups (usual-care vs. playlists without RAS vs. playlists with RAS).

The study utilized an intention to treat analysis. Categorical differences in baseline characteristics and outcomes were first evaluated between music and non-music groups incorporating Fishers’ exact test, or Mann-Whitney tests where appropriate. If significant differences between music and non-music groups existed, we then evaluated for differences in baseline characteristics and outcomes across all three groups, employing Fishers’ exact test and Kruskal-Wallis Test where appropriate.

Generalized Linear Modelling was used to fit the data using maximum likelihood equations (PROC GENMOD), and the first-order autoregressive function was used to examine weekly mean volume of physical activity across intervention groups, while accounting for repeated measures in weekly assessments.

Several sensitivity analyses were conducted which are described within the ‘Results’ section. Two-tailed *P* < 0.05 was defined as statistically significant. All statistical tests utilized SAS™ Version 9.3 (Cary, NC, USA).

## Results

### Baseline sample characteristics

The average age of the study sample was 63 years; 70% were male. Most patients were married, overweight and self-confident (i.e. high self-efficacy) in their abilities to self-manage cardiovascular disease and exercise. There were no significant differences in baseline physical activity volume between the music and non-music groups (Table 1) or across the three treatment arms (i.e. no-music, music without RAS, music with RAS) (Additional file 4).

**Table 1:** **Baseline characteristics of the study population**

	**No music**	**Music**	
	**Control**	**RAS/non-RAS combined**	***P*** **value** ^*^
	**(** ***n*** **= 11)**	**(** ***n*** **= 23)**	
Socio-demographic characteristics			
Age, mean years (STD)	66.4 (12.8)	61.5 (10.3)	0.25
Male (%)	6 (54.6)	17 (77.3)	0.24
Married (%)	7 (63.6)	20 (90.9)	0.15
Clinical factors			
Diabetes (%)	1 (9.1)	6 (30)	0.18
Past or current smoker (%)	5 (45.5)	12 (54.6)	0.62
Body mass index, mean (kg/m^2^) (STD)	27.7 (4.5)	28.6 (3.6)	0.56
Prior myocardial infarction (%)	2 (27.3)	5 (25)	0.89
Hypertension (%)	5 (45.5)	10 (50)	1.0
Lung disease (%)	2 (18.2)	4 (20)	0.90
Behavioural factors			
CES-Depression score, mean (STD)	6.25 (8.6)	9.6 (8.9)	0.62
Stanford self-efficacy, mean (STD)	80.6 (18.6)	86.5 (12.6)	0.37
Cardiac self-efficacy, mean (STD)	76.4 (19.4)	80.1 (14.9)	0.62
High behavioural risk^a^ (%)	5 (45.5)	12 (54.6)	1.0
Physical factors			
Baseline VO_2_, mean mL/kg/min (STD)	18.2 (5.7)	20.3 (5.0)	0.28
Week-1 volume of total activity, mean minutes (STD)	352.8 (408.5)	487.6 (379.6)	0.42
Week 1 volume of vigorous activity, mean minutes (STD)	1.45 (2.1)	5.4 (15.0)	0.98
Week 1 volume of moderate activity, mean minutes (STD)	105 (122.3)	162.3 (151.9)	0.31
Week 1 volume of light activity, mean minutes (STD)	246.4 (295.7)	320.7 (248.1)	0.68
Week 1 calorie burn, mean kcal (STD)	314.0 (359.4)	453.4 (366.9)	0.42

Baseline characteristics according to randomization group: No music controls vs. Music playlists (RAS and non-RAS combined). RAS, rhythmic auditory stimulation; STD, standard deviation. ^*^ *P* value tests for statistical difference between two groups (i.e. Control vs. Music-Playlist RAS/non-RAS combined); statistical tests for continuous variables used the Kruskal-Wallis Test, while statistical tests for categorical outcomes utilized Fisher’s Exact Test. ^a^Defined based on a BMI >30 kg/m^2^ or a CES-depression score of >15.

Non-significant intergroup difference in functional capacity and other baseline factors did exist. These were predominantly attributable to the recruitment of two outliers who were randomly allocated to the non-music usual-care control and to the RAS study arms where baseline VO_2peak_ among outliers resided at the extremes of fitness. One such patient was recruited to non-music usual-care, who was 82 years old and had a baseline VO_2peak_ of 8.9 mL∙kg∙min^−1^, while another patient, who was recruited to the music-playlist (with RAS) group, was 47 years old and had a baseline VO_2peak_ of 33.5 mL∙kg∙min^−1^.

Among the 34 participants, 3 patients dropped out of the study. Two were recruited to the music without RAS group and dropped out within 2 weeks of randomization (one patient never receiving any audio playlist device or activity monitor, while the other patient received both devices but was confirmed to have never used either the audio playlist device or activity monitor). One patient randomized to music playlists with RAS dropped out at week 5 of the study due to musculoskeletal limitations.

### Audio-playlist preferences and use

Table 2 represents the difference between music-playlists with RAS and without RAS enhancements. Patients randomized to playlists without RAS enhancement had slower walking pace and tempos of music, greater preferences towards contemporary (top 40 pop, hip-hop, dance), and fewer preferences for rock (classic and hard rock), jazz and classical music. The number of audio-plays was significantly higher among patients randomized to music playlists with RAS-enhancements than without (780.7 vs. 174.0 audio-plays, RAS vs. non-RAS groups, respectively, *P* < 0.001).

**Table 2:** **Music tempo-pace synchronization, preferences and utilization**

	**No music control**	**Music audio-playlist without RAS**	**Music audio-playlist with RAS**	***P*** **value** ^*^
	**(** ***n*** **= 11)**	**(** ***n*** **= 11)**	**(** ***n*** **= 11)**	
Playlist tempo-pace synchronization				
Exercise walking/running pace, mean steps/min (range)	N/A	110.7 (91 to 128.7)	122.0 (112 to 133)	0.03
Tempo range of music within playlist, mean BPM (range)	N/A	111 (81 to 139)	123 (105 to 168)	0.01
Patient playlist preferences				
Contemporary (e.g. top 40 pop, hip-hop, dance)	N/A	24%	11%	<0.001
Rock	N/A	18%	33%	
Country-Western	N/A	24%	17%	
Jazz, blues	N/A	6%	11%	
Classical	N/A	12%	22%	
Other (includes no stated preferences)	N/A	18%	6%	
Music-playlist utilization				
Average number of audio-plays	0	174 (186)	780.7 (943.7)	<0.001

According to randomized group: No music control vs. music audio-playlist without RAS vs. music audio-playlist with RAS enhancement_._ RAS, rhythmic auditory stimulation; BPM, beats per minute; N/A, not available. ^*^ *P* value tests for statistical differences across groups based on the Kruskal-Wallis Test or Chi-squared test.

Thematic content analysis of participant responses (*n* = 13) during the small group interviews found that 100% of the patients who used their audio-playlist devices identified music as being a key factor that helped them achieve their appropriate levels of intensity and duration of exercise. There were no differences in the perceived impact of music on exercise between RAS and non-RAS groups.

### Outcomes

**Figure 1:**
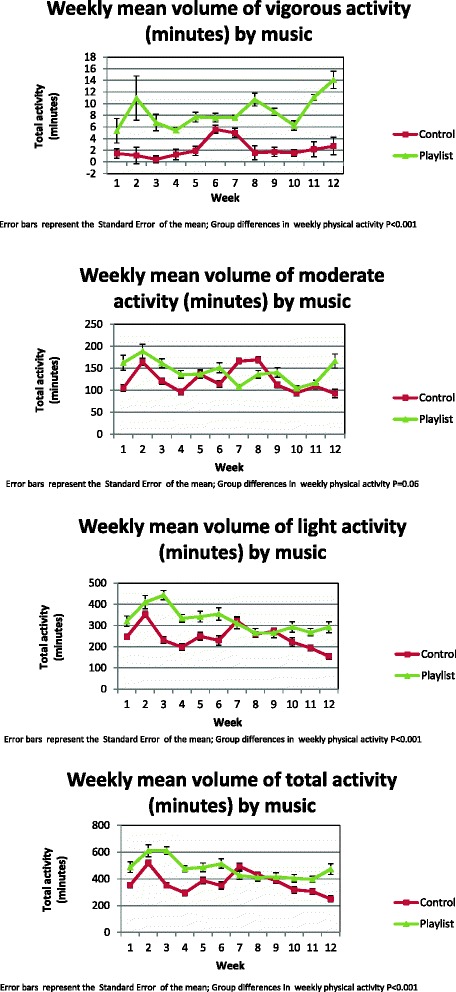
Weekly volume of physical activity, stratified by physical activity intensity. Vigorous activity (top graph); moderate activity (second from the top); light activity (third from the top); total activity (bottom graph). Non-music control (labelled ‘Control’: red line), music audio-playlist groups (irrespective of rhythmic auditory stimulation) (labelled ‘Playlist’: green line).

**Figure 2:**
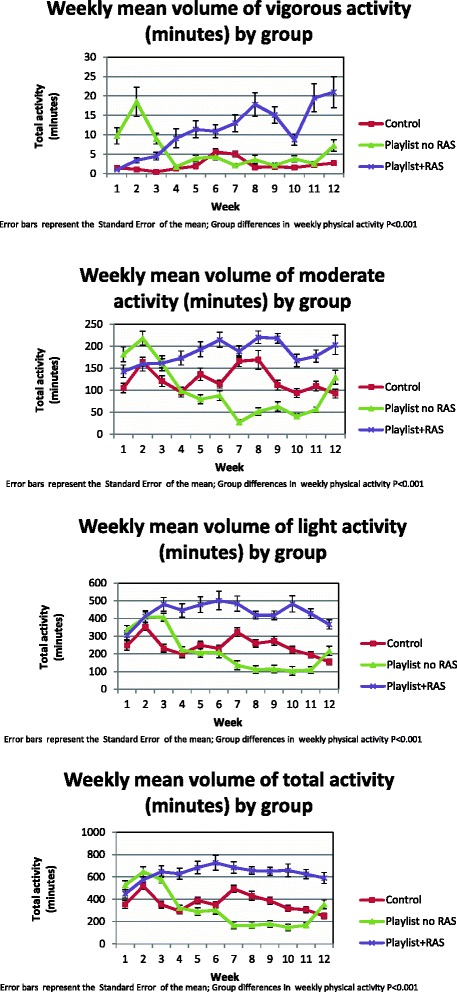
Weekly volume of physical activity, stratified by physical activity intensity. Vigorous activity (top graph); moderate activity (second from the top); light activity (third from the top); total activity (bottom graph). Non-music control (labelled ‘Control’: red line), music audio-playlist without RAS (labelled ‘Playlist’: green line), music audio-playlist with RAS (labelled ‘Playlist + RAS’: purple). RAS, rhythmic auditory stimulation.

**Figure 3:**
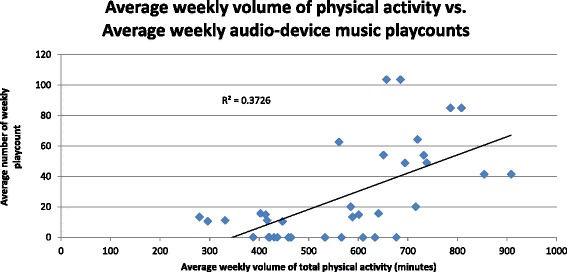
Average weekly volume of physical activity vs. average number of weekly audio-device music playcounts. The graph demonstrates the correlation between a group’s average weekly volume of physical activity (in minutes) and a group’s average number of weekly playcounts from the audio-playlist devices (*r* = 0.61, *P* < 0.001).

The weekly volume of total physical activity, as measured objectively using tri-axial accelerometer activity monitors, was higher among patients randomized to the group receiving personalized audio-playlists with tempo-pace synchrony as compared with non-music usual-care (Table 3, Figure 1). The improved physical activity volumes among patients randomized to personalized audio-playlists with tempo-pace synchrony was driven by the group receiving RAS-enhanced music playlists (average weekly minutes of physical activity of 631.3 min vs. 320 min vs. 370.2 min, music playlists with RAS vs. music playlists without RAS vs. usual-care, *P* < 0.001). Moreover, higher average weekly pertained to all intensity levels of physical activity (Table 4, Additional file 5) and was sustainable over time (Figure 2). The average number of weekly audio-plays per group strongly correlated with a group’s weekly average volume of physical activity (*r* = 0.61, *P* < 0.001), (Figure 3). There were no significant differences in weekly volumes of physical activity between the RAS unenhanced music group and the non-music usual-care controls, although this may have been explained by the limited use of audio-playlists among the non-RAS music arm.

**Table 3:** **Average weekly physical activity among those randomized to music playlists vs. No music control**

	**No music**	**Music**	
	**Control (** ***n*** **= 11)**	**RAS and no RAS combined (** ***n*** **= 22)**	***P*** **value** ^*^
Weekly estimated caloric expenditures, mean kcal (STD)	339.6 (307.2)	435.0 (407.4)	<0.001
Weekly volume of total activity, mean minutes (STD)	370.2 (332.5)	475.6 (446.0)	<0.001
Weekly volume of vigorous activity, mean minutes (STD)	2.22 (4.3)	8.5 (19.6)	<0.001
Weekly volume of moderate activity, mean minutes (STD)	123.3 (130.9)	142.3 (142.7)	0.06
Weekly volume of light activity, mean minutes (STD)	244.7 (230.7)	324.8 (326.3)	<0.001

^*^ *P* value tests for statistic difference between two groups (i.e. Music with or without RAS vs. Control). Weekly physical activity incorporated Generalized Linear Equations. Process and clinical outcomes incorporated the Kruskal-Wallis Test. Statistical tests for differences in categorical variables used Fishers’ Exact test. STD, standard deviation.

**Table 4:** **Average weekly physical activity volume**

	**No music control**	**Music audio-playlist without RAS**	**Music audio-playlist with RAS**	***P*** **value** ^*^
	**(** ***n*** **= 11)**	**(** ***n*** **= 11)**	**(** ***n*** **= 11)**	
Average weekly caloric expenditures, mean kcal (STD)	339.6 (307.2)	295.3 (327.8)	574.8 (431.9)	<0.001
Average weekly volume of total activity, mean minutes (STD)	370.2 (332.5)	320 (355.2)	631.3 (473.8)	<0.001
Average weekly volume of vigorous activity, mean minutes (STD)	2.22 (4.3)	5.7 (15.1)	11.3 (23.0)	<0.001
Average weekly volume of moderate activity, mean minutes (STD)	123.3 (130.9)	99.6 (120.4)	185.1 (150.7)	< 0.001
Average weekly volume of light activity, mean minutes (STD)	244.7 (230.7)	214.6 (254.7)	435 (352.6)	<0.001

According to randomized group: No music control vs. music audio-playlist without RAS vs. music audio-playlist with RAS enhancement. RAS, rhythmic auditory stimulation; STD, standard deviation. ^*^ *P* value tests for statistical differences across the three groups (i.e. Control vs. Playlist vs. Playlist-RAS enhanced). Self-managed activity incorporated Generalized Linear Equations. Process and clinical outcomes incorporated the Kruskal-Wallis Test. Statistical tests for differences in categorical variables used Fishers’ Exact test.

### Sensitivity analyses

Several sensitivity analyses were undertaken as follows:

First, all analyses using Generalized Longitudinal Models were repeated, accounting for variations within and between patients. Our results were similar. Namely, weekly total physical activity volume remained significantly higher among patients randomized to the music intervention, especially among those with RAS-enhancement (dose-response differences across the three groups of 121.6 min of weekly total activity, *P* = 0.04). Moreover, the relationship persisted among intensity-specific physical activity, including vigorous physical activity (dose response differences across the three groups of 4.5 min of weekly vigorous activity, *P* = 0.08).

Second, the positive relationship between music intervention and physical activity adherence remained significant among high-risk subgroups (defined *a priori* based on a BMI exceeding 30 kg/m^2^ and a CES-Depression score exceeding 15 [37]) and did so irrespective of the employment of different thresholds for risk and/or the inclusion of individual’s predicted likelihood of program drop-out based on on-site attendance (Additional file 6).

Third, to determine when differences in weekly physical activity volume emerged over time, we subdivided the weekly intervals into three equivalent time intervals (weeks 0 to 4; weeks 5 to 8; weeks 9 to 12) and repeated the analyses accordingly. Significant group-specific differences in physical activity diverged and become statistically significant only after week 5, (i.e. group-specific differences during weeks 0 to 4, weeks 5 to 8, and weeks 9 to 12, *P* = 0.22, *P* = 0.0004, *P* < 0.0001, respectively), suggesting that improvements in physical activity behaviours associated with RAS were sustainable.

Finally, to explore whether differences in baseline characteristics explained group-differences in outcomes, two additional sensitivity analyses were performed. The first analysis adjusted for baseline characteristics by forcing baseline covariates into the model. Group effects in total weekly activity remained significant, even after adjusting for age, marital status and baseline cardiopulmonary fitness (*P* = 0.05) - group-specific differences in baseline characteristics which were most imbalanced despite randomization. The second excluded the two patient outliers described above whose baseline VO_2peak_ fell at the extremes (8.9 and 33.5 mL/kg/min). The removal of these two patients resulted in similar VO_2peak_ across the three groups (19.1 mL∙kg∙min^−1^ vs. 19.1 mL∙kg∙min^−1^ vs. 19.8 mL∙kg∙min^−1^ in the control group vs. music without RAS vs. music playlist with RAS, respectively) and did not meaningfully alter our results. Group differences in physical activity still favored the group receiving music audio-playlists with RAS (*P* = 0.02).

## Discussion

Our study demonstrated the feasibility and efficacy of implementing a tempo-pace synchronized preference-based audio-playlist strategy to improve adherence to physical activity among patients receiving cardiac rehabilitation. Patients randomized to personalized audio-playlists with tempo-pace synchronized music embarked on significantly greater amounts of weekly physical activity and experienced lower declines in weekly physical activity than did their non-music usual-care counterparts. Differences in physical activity among those randomized to music playlists were driven predominantly by the group randomized to RAS, where attainment in weekly physical activity volumes were twofold higher than those randomized to music but without RAS enhancements. Improvements in the weekly volume of physical activity associated with RAS increased over time, were sustainable, applied to all intensity-levels of physical activity and were as pronounced among high-risk as it was among low-risk subgroups.

Our study builds on existing music-exercise literature in several additional ways. First, to the best of our knowledge, ours is the first study to have examined the longitudinal adherence-effects of an exercise-music strategy within a structured exercise program. Until now, the vast majority of studies exploring the effects of music in exercise have been cross-sectional in design and/or narrow in outcome ascertainment, with most studies limited to ergonomic and/or perceptual outcomes [8-16,44-46]. Second, we employed a novel integrated audio-playlist strategy where patient preferences informed the choices of musical genres and artists but where exercise pace restricted the playlist selection to those songs whose music tempos approximated individuals’ program-prescribed steps per minute. Third, our study demonstrated that RAS-enhancements, which were designed to accentuate the auditory cues associated with tempo-pace synchrony [24,27], were accomplished feasibly using commercially available customized playlists. Moreover, such RAS-enhanced playlists were incrementally efficacious in improving long-term physical activity behaviours. Finally, ours, to the best of our knowledge, is the first to compare the behavioural outcomes associated with a personalized music strategy against an evidence-based gold-standard usual-care control, which itself, has been associated with a 20% improvement in long-term survival [4].

Available evidence suggests that tempo-pace synchronous music can reduce exercise-perceived exertion, increase exercise endurance, augment exercise intensity and extend the duration of exercise [19,21,26,27,31-33]. The effects of synchronized music on exercise function have been shown to be greater when music is aligned with individual music preferences. Such alignment can augment affect and enhance intrinsic motivation [31,32,47-49]. Affect augmentation along with the incorporation of other self-regulatory behavioural self-management techniques (i.e. goal-setting, self-monitoring) [50] may have narrowed the intention-motivation-behavioural gap associated with exercise through improved automaticity and organization [51]. While there is supportive evidence suggesting that the combination of affect augmentation with rhythmic synchronization could impact physical activity behaviour [11,21,23,52,53], no study had ever directly tested this hypothesis until now.

Alternate explanations for higher weekly physical activity volumes among those randomized to RAS may have existed. Most importantly, patients randomized to tempo-pace synchronized music with RAS enhancements listened to their audio-devices nearly fourfold more than those who received tempo-pace audio-music devices without RAS enhancements. The correlation between average weekly audio-playcounts and average weekly physical activity volumes precluded us from disentangling the efficacy of audio music playlist devices in general, from tempo-pace synchrony or RAS.

The feasibility of integrating a tempo-pace synchronization strategy into commercially available music in a manner which takes into account personal patient preferences underscores the *potential* for ‘real-world’ application. All songs comprising an individual’s personalized playlist contained tempos that were calibrated to within 10 beats per minute of a patient’s prescribed exercise pace - a calibration that falls within ‘tempo-pace’ asynchronous thresholds where stride length can be modified to accommodate changes in music tempo without perceptual differences in effort [18]. The tempos of commercially available music were ascertained using widely available automated software application tools that are widely available on the web. The implementation of RAS sonic-enhancements to commercially available music was also implemented in a feasible manner by embedding digital auditory stimuli into the existing original sound recordings using MIDI keyboard instruments and commercially available recording software. The original audio-files and the superimposed digital stimuli were then re-mixed into one composite sound recording. One challenge encountered was the fact that the tempos of the original sound recording (i.e. music files within an individual’s audio-playlist) were rarely recorded in quantized fashion. As a result, small fluctuations in the tempo of the original recordings existed. To overcome this limitation, sonic enhancements were added manually and played along in real time with the original audio-recording. Additional manual modifications allowed us to optimize the tempo-synchrony between RAS and the original sound music recording where necessary. Future research will need to explore how such methodology can be better automated and standardized.

Our study was designed as a proof-of-principle feasibility study. However, there were several noteworthy study limitations that deserve consideration. First, the sample size of our study was small. Second, with the exception of RAS enhancements which did incorporate patient blinding, the study was largely un-blinded. Third, despite our randomized design, imbalances in baseline characteristics existed across groups. While such imbalances were not statistically significant per se, it is possible that residual confounding may have existed and accounted for some or all of our results. Finally, differences in music preferences, genres and music tempos may have partially explained the variations in exercise adherence between RAS and non-RAS groups [18,54,55].

Notwithstanding such caveats, we believe our findings justify the need for further research through rigorously designed clinical trials. If reproducible, such findings could have potentially transformative health implications. For example, the average difference of 105.4 min in weekly physical activity between those receiving and not receiving music playlists, if sustained, would correlate with a projected life-expectancy gain of 2.5 years for an average male 65-year-old patient [56,57]. Given that benefits of music playlists were equally applicable among those patients who were at higher risk of cardiac rehabilitation programmatic drop-out than those at lower behavioural attrition risk, the absolute survival benefits may be even greater among those most behaviourally disengaged. With their modest associated costs, the use of preference-based music audio playlist devices (i.e. approximately $75 per patient in our study) could also have important cost-effectiveness implications as a health-policy strategy to improve physical activity adherence in the population [58,59].

## Conclusions

In conclusion, our results demonstrated the integration of personalized music playlists with tempo-pace synchronization into a structured exercise program was feasible and efficacious at improving adherence to physical activity. Those randomized to audio-music playlists with RAS enhancements attained significantly greater weekly volumes of physical activity than the evidence-based non-music usual standard of care. Such findings may have important clinical application when attempting to engage otherwise behaviourally disengaged populations into structured physical activity programs. Further research is required to reproduce such findings in larger real-world settings.

## Additional files

Additional file 1:
**Supplemental appendix 1.** Percentage distribution of consenting males and females per cardiac rehabilitation class. Additional file 2:
**Supplemental appendix 2.** Examples of the visual display of sound frequencies and their respective intensities within the sonic audio WAV files without and with RAS enhancements. Additional file 3:
**Supplemental appendix 3.** Sonic attributes associated RAS enhancements. Additional file 4:
**Supplemental appendix 4.** Baseline characteristics of the study population according to randomization group (No music control vs. music audio-playlist without RAS vs. music audio-playlist with RAS enhancement). Additional file 5:
**Supplemental appendix 5.** Test statistics for three-way comparisons between non-music usual-care control vs. audio-playlist without RAS vs. audio-playlist with RAS. Additional file 6:
**Supplemental appendix 6.** The positive relationship between music intervention and physical activity adherence remained significant among high-risk subgroups.

## References

[1] Woodcock J, Franco OH, Orsini N, Roberts I (2011). Non-vigorous physical activity and all-cause mortality: systematic review and meta-analysis of cohort studies. Int J Epidemiol.

[2] Nocon M, Hiemann T, Muller-Riemenschneider F, Thalau F, Roll S, Willich SN (2008). Association of physical activity with all-cause and cardiovascular mortality: a systematic review and meta-analysis. Eur J Cardiovasc Prev Rehabil.

[3] Colley RC, Garriguet D, Janssen I, Craig CL, Clarke J, Tremblay MS (2011). Physical activity of Canadian adults: accelerometer results from the 2007 to 2009 Canadian Health Measures Survey. Health Rep.

[4] Heran BS, Chen JM, Ebrahim S, Moxham T, Oldridge N, Rees K, Thompson DR, Taylor RS, Exercise-based cardiac rehabilitation for coronary heart disease. Cochrane Database Syst Rev. 2011;7:CD001800. 10.1002/14651858.CD001800.pub2 PMC4229995 21735386

[5] West RR, Jones DA, Henderson AH (2012). Rehabilitation after myocardial infarction trial (RAMIT): multi-centre randomised controlled trial of comprehensive cardiac rehabilitation in patients following acute myocardial infarction. Heart.

[6] Davies P, Taylor F, Beswick A, Wise F, Moxham T, Rees K, Ebrahim S (2010). Promoting patient uptake and adherence in cardiac rehabilitation. Cochrane Database Syst Rev.

[7] Chase JA (2011). Systematic review of physical activity intervention studies after cardiac rehabilitation. J Cardiovasc Nurs.

[8] Yamashita S, Iwai K, Akimoto T, Sugawara J, Kono I (2006). Effects of music during exercise on RPE, heart rate and the autonomic nervous system. J Sports Med Phys Fitness.

[9] Anshel MH, Marisi D (1978). Effect of music and rhythm on physical performance. Res Q.

[10] Cervellin G, Lippi G (2011). From music-beat to heart-beat: a journey in the complex interactions between music, brain and heart. Eur J Intern Med.

[11] Clark IN, Taylor NF, Baker F (2012). Music interventions and physical activity in older adults: a systematic literature review and meta-analysis. J Rehabil Med.

[12] Karageorghis CI, Mouzourides DA, Priest DL, Sasso TA, Morrish DJ, Walley CJ (2009). Psychophysical and ergogenic effects of synchronous music during treadmill walking. J Sport Exerc Psychol.

[13] Fritz TH, Hardikar S, Demoucron M, Niessen M, Demey M, Giot O, Li Y, Haynes JD, Villringer A, Leman M, Musical agency reduces perceived exertion during strenuous physical performance. Proc Natl Acad Sci U S A. 2013;110(44):17784–9. 10.1073/pnas.1217252110 PMC3816438 24127588

[14] Karageorghis C, Jones L, Stuart DP (2008). Psychological effects of music tempi during exercise. Int J Sports Med.

[15] Simpson SD, Karageorghis CI (2006). The effects of synchronous music on 400-m sprint performance. J Sports Sci.

[16] Terry PC, Karageorghis CI, Saha AM, D’Auria S (2012). Effects of synchronous music on treadmill running among elite triathletes. J Sci Med Sport.

[17] Thaut MH, Kenyon GP, Schauer ML, McIntosh GC (1999). The connection between rhythmicity and brain function. IEEE Eng Med Biol Mag.

[18] Leman M, Moelants D, Varewyck M, Styns F, van NL Martens JP (2013). Activating and relaxing music entrains the speed of beat synchronized walking. PLoS One.

[19] Khalfa S, Roy M, Rainville P, Dalla BS, Peretz I (2008). Role of tempo entrainment in psychophysiological differentiation of happy and sad music?. Int J Psychophysiol.

[20] Potteiger JA, Schroeder JM, Goff KL (2000). Influence of music on ratings of perceived exertion during 20 minutes of moderate intensity exercise. Percept Mot Skills.

[21] van der Vlist B, Bartneck C, Maueler S (2011). moBeat: using interactive music to guide and motivate users during aerobic exercising. Appl Psychophysiol Biofeedback.

[22] Molinari M, Leggio MG, De MM, Cerasa A, Thaut M (2003). Neurobiology of rhythmic motor entrainment. Ann N Y Acad Sci.

[23] Lima-Silva AE, Silva-Cavalcante MD, Pires FO, Bertuzzi R, Oliveira RS, Bishop D (2012). Listening to music in the first, but not the last 1.5 km of a 5-km running trial alters pacing strategy and improves performance. Int J Sports Med.

[24] Mendonca C, Oliveira M, Fontes L, Santos J (2014). The effect of instruction to synchronize over step frequency while walking with auditory cues on a treadmill. Hum Mov Sci.

[25] Sejdic E, Jeffery R, Vanden KA, Chau T (2012). An investigation of stride interval stationarity while listening to music or viewing television. Hum Mov Sci.

[26] Creel SC (2012). Similarity-based restoration of metrical information: different listening experiences result in different perceptual inferences. Cogn Psychol.

[27] Honing H (2012). Without it no music: beat induction as a fundamental musical trait. Ann N Y Acad Sci.

[28] Kenyon GP, Thaut MH, Thaut MH (2008). Rhythm-driven optimization of motor control. Rhythm, Music, and the brain: scientific foundations and clinical applications.

[29] Will U, Berg E (2007). Brain wave synchronization and entrainment to periodic acoustic stimuli. Neurosci Lett.

[30] Nozaradan S, Peretz I, Mouraux A (2012). Selective neuronal entrainment to the beat and meter embedded in a musical rhythm. J Neurosci.

[31] Nakamura PM, Pereira G, Papini CB, Nakamura FY, Kokubun E (2010). Effects of preferred and nonpreferred music on continuous cycling exercise performance. Percept Mot Skills.

[32] Macnay SK (1995). The influence of preferred music on the perceived exertion, mood, and time estimation scores of patients participating in a cardiac rehabilitation exercise program. Mus Ther Perspect.

[33] Popescu M, Otsuka A, Ioannides AA (2004). Dynamics of brain activity in motor and frontal cortical areas during music listening: a magnetoencephalographic study. Neuroimage.

[34] Hammill BG, Curtis LH, Schulman KA, Whellan DJ (2010). Relationship between cardiac rehabilitation and long-term risks of death and myocardial infarction among elderly Medicare beneficiaries. Circulation.

[35] Alter DA, Zagorski B, Marzolini S, Forhan M, Oh PI. On-site programmatic attendance to cardiac rehabilitation and the healthy-adherer effect. Eur J Prev Cardiol. 2014. doi: 10.1177/2047487314544084. 10.1177/2047487314544084 25079239

[36] Hogan T, Sundaram M (1989). Rhythmic auditory stimulation in generalized epilepsy. Electroencephalogr Clin Neurophysiol.

[37] Forhan M, Zagorski BM, Marzonlini S, Oh P, Alter DA (2013). Predicting exercise adherence for patients with obesity and diabetes referred to a cardiac rehabilitation and secondary prevention program. Can J Diabetes.

[38] Cadence software. http://www.cadenceappcom/2014.

[39] Chen JL, Zatorre RJ, Penhune VB (2006). Interactions between auditory and dorsal premotor cortex during synchronization to musical rhythms. Neuroimage.

[40] Beekman AT, Deeg DJ, Van LJ, Braam AW, De Vries MZ, Van TW (1997). Criterion validity of the Center for Epidemiologic Studies Depression scale (CES-D): results from a community-based sample of older subjects in The Netherlands. Psychol Med.

[41] Lorig KR, Sobel DS, Ritter PL, Laurent D, Hobbs M (2001). Effect of a self-management program on patients with chronic disease. Eff Clin Pract.

[42] Hedeker D, Gibbons RD, Waternaux C (1999). Sample size estimation for longitudinal designs with attrition. J Educ Behav Stat.

[43] Slootmaker SM, Chin A Paw JM, Schuit AJ, van Mechelen W, Koppes LL. Concurrent validity of the PAM accelerometer relative to the MTI Actigraph using oxygen consumption as a reference. Scand J Med Sci Sports. 2009;19(1):36–43. 10.1111/j.1600-0838.2007.00740.x 18266793

[44] Brownley KA, McMurray RG, Hackney AC (1995). Effects of music on physiological and affective responses to graded treadmill exercise in trained and untrained runners. Int J Psychophysiol.

[45] Hayakawa Y, Miki H, Takada K, Tanaka K (2000). Effects of music on mood during bench stepping exercise. Percept Mot Skills.

[46] Lim HB, Atkinson G, Karageorghis CI, Eubank MR (2009). Effects of differentiated music on cycling time trial. Int J Sports Med.

[47] Bellebaum C, Koch B, Schwarz M, Daum I (2008). Focal basal ganglia lesions are associated with impairments in reward-based reversal learning. Brain.

[48] Zatorre RJ, Salimpoor VN (2013). From perception to pleasure: music and its neural substrates. Proc Natl Acad Sci U S A.

[49] Karageorghis CI, Priest DL (2012). Music in the exercise domain: a review and synthesis (Part I). Int Rev Sport Exerc Psychol.

[50] Ferrier S, Blanchard CM, Vallis M, Giacomantonio N (2011). Behavioural interventions to increase the physical activity of cardiac patients: a review. Eur J Cardiovasc Prev Rehabil.

[51] Rhodes RE (2014). Bridging the physical activity intention-behaviour gap: contemporary strategies for the clinician. Appl Physiol Nutr Metab.

[52] Mangeri F, Montesi L, Forlani G, Dalle GR, Marchesini G (2014). A standard ballroom and Latin dance program to improve fitness and adherence to physical activity in individuals with type 2 diabetes and in obesity. Diabetol Metab Syndr.

[53] Cho J. The effect of music therapy on mood, perceived exertion, and exercise adherence of patients participating in a rehabilitative upper extremity exercise program. University of Kansas, Master’s Thesis: Music Education & Music Therapy. 2009. https://kuscholarworks.ku.edu/bitstream/handle/1808/5529/Cho_ku_0099M_10419_DATA_1.pdf?sequence=1. Accessed April 29, 2015.

[54] Edworthy J, Waring H (2006). The effects of music tempo and loudness level on treadmill exercise. Ergonomics.

[55] Karageorghis CI, Terry PC, Lane AM, Bishop DT, Priest DL (2012). The BASES Expert Statement on use of music in exercise. J Sports Sci.

[56] Wen CP, Wai JP, Tsai MK, Yang YC, Cheng TY, Lee MC, Chan HT, Tsao CK, Tsai DS, Wu X, Minimum amount of physical activity for reduced mortality and extended life expectancy: a prospective cohort study. Lancet. 2011;378(9798):1244–53. 10.1016/S0140-6736(11)60749-6 21846575

[57] Franco OH, de Laet C, Peeters A, Jonker J, Mackenbach J, Nusselder W. Effects of physical activity on life expectancy with cardiovascular disease. Arch Intern Med. 2005;165(20):2355–60. 10.1001/archinte.165.20.2355 16287764

[58] Moy ML, Teylan M, Weston NA, Gagnon DR, Garshick E (2013). Daily step count predicts acute exacerbations in a US cohort with COPD. PLoS One.

[59] LaCroix AZ, Leveille SG, Hecht JA, Grothaus LC, Wagner EH (1996). Does walking decrease the risk of cardiovascular disease hospitalizations and death in older adults?. J Am Geriatr Soc.

