# Supplementary material for: Synchronized personalized music audio-playlists to improve adherence to physical activity among patients participating in a structured exercise program: a proof-of-principle feasibility study
Source: Sports Med Open. 2015 May 8;1:23. doi: 10.1186/s40798-015-0017-9 (PMC5005752; doi:10.1186/s40798-015-0017-9)
Supplement: Additional file 1: — Supplemental appendix 1. Percentage distribution of consenting males and females per cardiac rehabilitation class. [file 40798_2015_17_MOESM1_ESM.docx]

**Supplemental Appendix Table 1:** Percentage distribution of consenting males and females per cardiac rehabilitation class

|  | **% Males** | **% Females** | **% Male consented** | **% Female consented** |
| --- | --- | --- | --- | --- |
| **Class 1** | 79 | 21 | 100 | 0 |
| **Class 2** | 77 | 23 | 66 | 33 |
| **Class 3** | 79 | 21 | 100 | 0 |
| **Class 4** | 62 | 38 | 100 | 0 |
| **Class 5** | 85 | 15 | 75 | 25 |
| **Class 6** | 75 | 25 | 75 | 25 |
| **Class 7** | 65 | 35 | 63 | 37 |
| **Class 8** | 76 | 24 | 57 | 43 |
|  |  |  |  |  |
