# Supplementary material for: Synchronized personalized music audio-playlists to improve adherence to physical activity among patients participating in a structured exercise program: a proof-of-principle feasibility study
Source: Sports Med Open. 2015 May 8;1:23. doi: 10.1186/s40798-015-0017-9 (PMC5005752; doi:10.1186/s40798-015-0017-9)
Supplement: Additional file 2: — Supplemental appendix 2. Examples of the visual display of sound frequencies and their respective intensities within the sonic audio WAV files without and with RAS enhancements. [file 40798_2015_17_MOESM2_ESM.docx]

**Supplemental Appendix 2** : Examples of the visual display of sound-frequencies and their respective intensities within the sonic audio WAV files without and with RAS enhancements.

**Example 1:**

**Song 1 without RAS (0-12 seconds)**


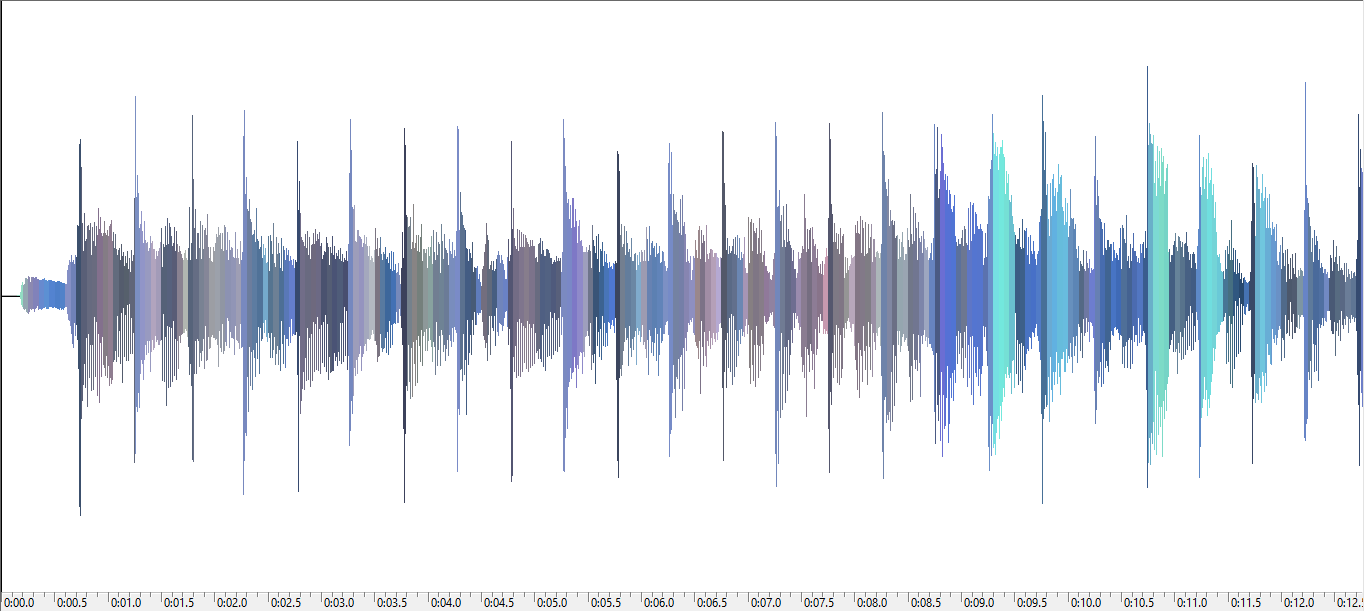


Intensity (Db)

**121.61 Hz**

**Song 1 with RAS (0-12 seconds)**


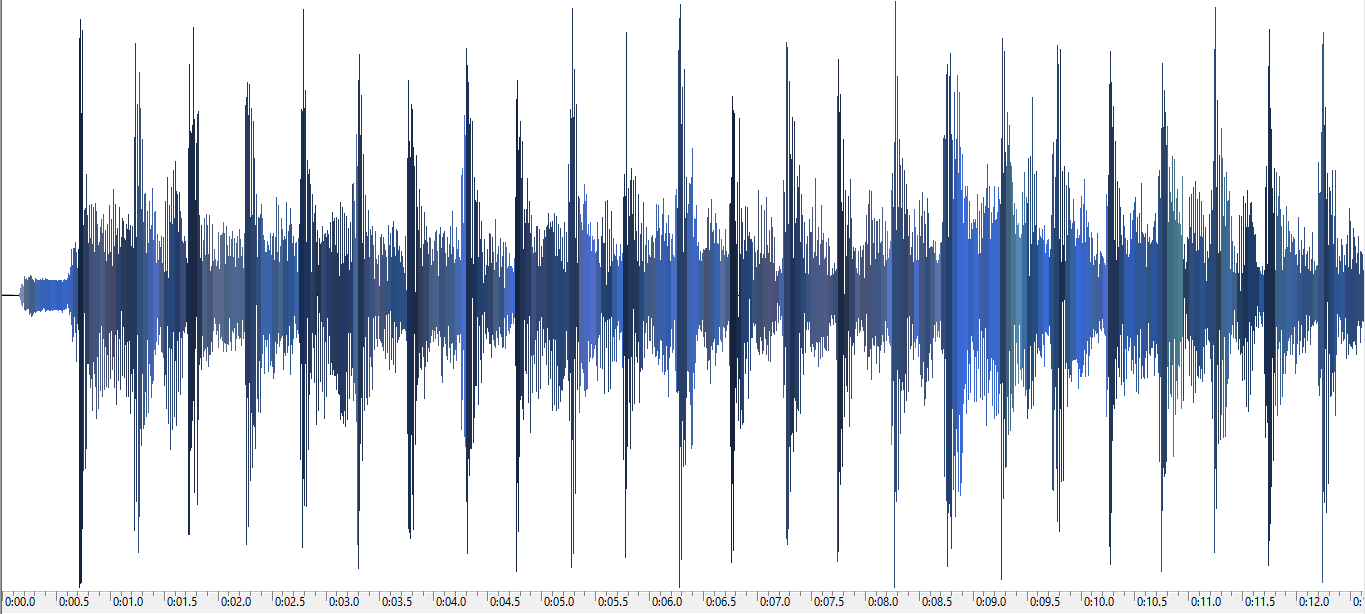


Greatly increased maximum amplitude of the rhythmic RAS components relative to the remaining frequencies of the sound-recording

Lower frequency of peaks

Intensity (Db)

**Song 2 without RAS (0-12 seconds)**

**67.33 Hz**

**Example 2:**


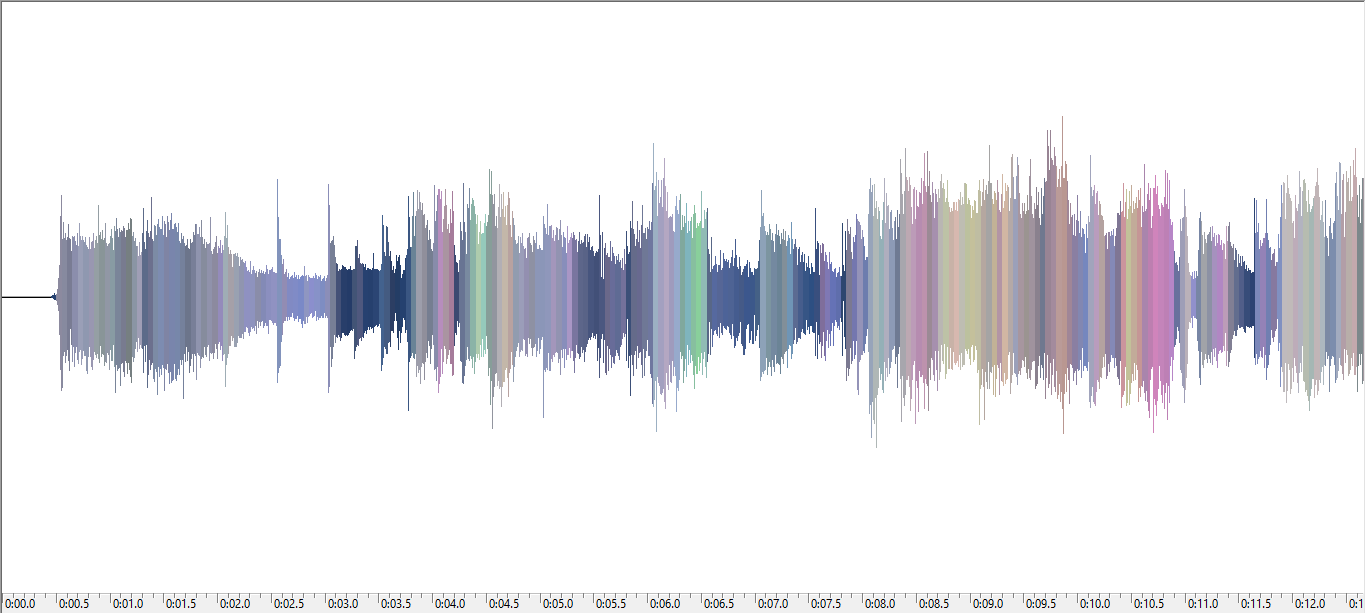


**Song 2 with RAS (0-12 seconds)**

Intensity (Db)


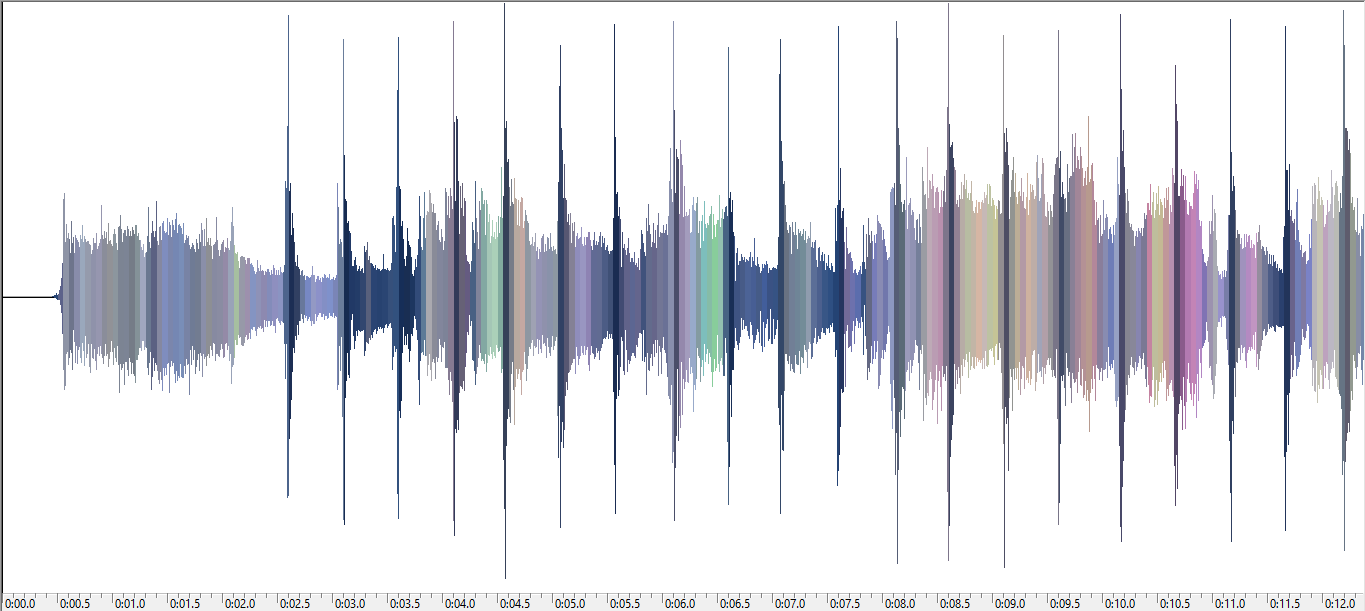


Greatly increased maximum amplitude of the rhythmic RAS components relative to the remaining frequencies of the sound-recording

Lower frequency of peaks

Increased number of peaks in amplitude, and increased slope of peaks

84 Hz

207.78 Hz

Intensity (Db)
