# Supplementary material for: Synchronized personalized music audio-playlists to improve adherence to physical activity among patients participating in a structured exercise program: a proof-of-principle feasibility study
Source: Sports Med Open. 2015 May 8;1:23. doi: 10.1186/s40798-015-0017-9 (PMC5005752; doi:10.1186/s40798-015-0017-9)
Supplement: Additional file 3: — Supplemental appendix 3. Sonic attributes associated RAS enhancements. [file 40798_2015_17_MOESM3_ESM.docx]

**Supplemental Appendix 3.** Sonic attributes associated RAS enhancements.^[[1]](#endnote-1)^

|  | **Music audio-playlist with RAS-enhancements**  **(n=11)** | |  |
| --- | --- | --- | --- |
|  |  |  | **P value^[[2]](#endnote-2)^** |
|  |  |  |  |
|  | **RAS unenhanced** | **RAS enhanced** |  |
| Peak frequencies per second of songs in playlist (STD) | 1.38 (0.64) | 1.91 (0.36) | <0.001 |
| Peak volumes relative to baseline volumes in playlist (STD) | 10.4 (2.9) | 13 (2.6) | 0.0003 |
| Frequency level at peak-frequencies in playlist (STD) | 159.4 (56.9) | 118.0 (57.3) | 0.02 |
| Slope of peak frequencies in playlist (STD) | 523.9 (399.3) | 719.0 (354.3) | 0.03 |
|  |  |  |  |
|  |  |  |  |

1. For this group of patients, the music included in this playlist included both RAS enhanced and unenhanced music. Patients were not told which music was RAS enhanced. RAS=Rhythmic Auditory Stimulation. The analysis was conducted on the first 28 consecutive songs used in the RAS group. [↑](#endnote-ref-1)
2. P-value tests for statistical differences across groups based on the Kruskal-Wallis Test or Chi-squared test. [↑](#endnote-ref-2)
