# Supplementary material for: Synchronized personalized music audio-playlists to improve adherence to physical activity among patients participating in a structured exercise program: a proof-of-principle feasibility study
Source: Sports Med Open. 2015 May 8;1:23. doi: 10.1186/s40798-015-0017-9 (PMC5005752; doi:10.1186/s40798-015-0017-9)
Supplement: Additional file 4: — Supplemental appendix 4. Baseline characteristics of the study population according to randomization group (No music control vs. music audio-playlist without RAS vs. music audio-playlist with RAS enhancement). [file 40798_2015_17_MOESM4_ESM.docx]

**Supplemental Appendix 4.** Baseline characteristics of the study population according to randomization group (No music control vs. music audio-playlist without RAS vs. music audio-playlist with RAS enhancement).^[[1]](#endnote-1)^

|  | **No music control**  (n=11) | **Music audio-playlist without RAS**  (n=12) | **Music audio-playlist with RAS**  (n=11) | **P value^[[2]](#endnote-2)^** |
| --- | --- | --- | --- | --- |
| **Socio-demographic factors** |  |  |  |  |
| Age, mean years (STD^[[3]](#endnote-3)^) | 66.4 (12.8) | 57.9 (10.6) | 65.9 (8.3) | 0.09 |
| Male, % | 6 (54.6) | 8 (66.7) | 10 (90.9) | 0.19 |
| Married, %  **Clinical factors** | 7 (63.6) | 9 (83.3) | 11 (100) | 0.09 |
| Diabetes, % | 1 (9.1) | 2 (20) | 4 (40) | 0.24 |
| Past or current smoker,% | 5 (45.5) | 5 (41.7) | 7 (63.6) | 0.69 |
| Body Mass Index, mean (kg/m2) (STD^‡^) | 27.7 (4.5) | 28.5 (3.7) | 28.9 (3.7) | 0.61 |
| Prior Myocardial Infarction, % | 2 (27.3) | 2 (20) | 3 (30) | 0.90 |
| Hypertension, % | 5 (45.5) | 6 (60.0) | 4 (40.0) | 0.74 |
| Lung disease, % | 2 (18.2) | 1 (10) | 3 (30) | 0.52 |
| **Behavioural factors** |  |  |  |  |
| CES-Depression score, mean (STD^‡^) | 6.25 (8.6) | 10.8 (10.4) | 8.4 (7.4) | 0.56 |
| Stanford self-efficacy, mean (STD^‡^) | 80.6 (18.6) | 84.2 (14.9) | 88.8 (10.4) | 0.64 |
| Cardiac self-efficacy, mean (STD^‡^) | 76.4 (19.4) | 75.8 (16.4) | 83.9 (13.4) | 0.53 |
| High behavioral risk,^[[4]](#endnote-4)^ % | 5 (45.5) | 7 (63.6) | 5 (45.5) | 1.0 |
| **Physical activity** **measures** |  |  |  |  |
| Baseline VO2, mean ml/kg/min (STD^‡^) | 18.2 (5.7) | 19.1 (4.1) | 21.1 (5.9) | 0.46 |
| Week-1 volume of total activity, mean minutes (STD^‡^) | 352.8 (408.5) | 526.5 (398.6) | 448.7 (376.7) | 0.56 |
| Week-1 volume of light activity, mean minutes (STD^‡^) | 246.4 (295.7) | 335.4 (248.9) | 304.6 (248.9) | 0.61 |
| Week-1 volume of moderate activity, mean minutes (STD^‡^) | 105.0 (122.3) | 181.4 (165.4) | 143.1 (143.2) | 0.53 |
| Week-1 volume of vigorous activity, mean minutes (STD^‡^) | 1.5 (2.1) | 9.7 (20.8) | 1.4 (1.4) | 0.97 |
| Week-1 calorie burn, mean Kcal (STD^‡^) | 314.0 (359.4) | 498.3 (399.8) | 404.5 (350.1) | 0.56 |

1. RAS = Rhythmic Auditory Stimulation [↑](#endnote-ref-1)
2. P-value tests for statistical differences across the 3 groups (i.e., Control vs. Playlist vs. Playlist-RAS enhanced); statistical tests for continuous outcome variables used the Kruskal-Wallis Test, while statistical tests for categorical outcomes utilized Fisher’s Exact test. [↑](#endnote-ref-2)
3. STD = Standard Deviation [↑](#endnote-ref-3)
4. Defined based on a BMI>30 kg/m2 or a CES-depression score of >15. [↑](#endnote-ref-4)
