# Supplementary material for: Synchronized personalized music audio-playlists to improve adherence to physical activity among patients participating in a structured exercise program: a proof-of-principle feasibility study
Source: Sports Med Open. 2015 May 8;1:23. doi: 10.1186/s40798-015-0017-9 (PMC5005752; doi:10.1186/s40798-015-0017-9)
Supplement: Additional file 5: — Supplemental appendix 5. Test statistics for three-way comparisons between non-music usual-care control vs. audio-playlist without RAS vs. audio-playlist with RAS. [file 40798_2015_17_MOESM5_ESM.docx]

**Supplemental Appendix 5:** Test statistics for 3 way comparisons between non-music usual care control vs. audio-playlist without RAS vs. audio-playlist with RAS

| **Test statistics for Supplemental Appendix 5** |  |  |  |
| --- | --- | --- | --- |
| *Variables testing across all 3 groups* | *Kruskal-Wallis*  *Chi-square* | *Degree of Freedom* | *P valve* |
| Age | 4.097 | 2 | 0.13 |
| Cardiac self-efficacy | 1.27 | 2 | 0.53 |
| Stanford self-efficacy | 0.897 | 2 | 0.64 |
| Baseline peak VO2 | 1.17 | 2 | 0.56 |
| CES (depression score) | 0.393 | 2 | 0.82 |
| BMI | 0.99 | 2 | 0.61 |
| Week 1 calorie burn | 0.975 | 2 | 0.61 |
| Week 1 Vigorous activity | 0.701 | 2 | 0.70 |
| Week 1 moderate activity | 1.33 | 2 | 0.51 |
| Week 1 Light activity | 0.79 | 2 | 0.67 |
| Week 1Total activity | 0.795 | 2 | 0.67 |
| **Test statistics for table 5** |  |  |  |
| *Variable testing across all 3 groups* | *Kruskal-Wallis*  *Chi-square* | *Degree of Freedom* | *P valve* |
| Average weekly energy burn | 32.64 | 2 | <0.0001 |
| Average weekly vigorous activity | 5.27 | 2 | 0.07 |
| Average weekly moderate activity | 25.89 | 2 | <0.0001 |
| Average weekly light activity | 34.2 | 2 | <0.0001 |
| Average weekly total activity | 33.6 | 2 | <0.0001 |
| **2-way comparisons Wilcoxn-Mann-Whitney** |  |  |  |
| *Non RAS audio-playlists vs. RAS audio-playlists* | *Wilcoxon statistic* | *Z score* |  |
| Average weekly energy burn | 11629.6 | -5.3 | <0.0001 |
| Average weekly vigorous activity | 13417 | -2.22 | 0.03 |
| Average weekly moderate activity | 11835 | -4.9 | <0.0001 |
| Average weekly light activity | 11621.5 | -5.3 | <0.0001 |
| Average weekly total activity | 11621.5 | -5.3 | <0.0001 |
| *RAS audio-playlists vs. no music usual-care* | *Wilcoxon statistic* | *Z score* |  |
| Average weekly energy burn | 17690.0 | 4.36 | <0.0001 |
| Average weekly vigorous activity | 15912.0 | 1.39 | 0.16 |
| Average weekly moderate activity | 17179.5 | 3.47 | 0.0005 |
| Average weekly light activity | 17854.0 | 4.65 | <0.0001 |
| Average weekly total activity | 17779.0 | 4.51 | <0.0001 |
| *No music usual-care vs. non RAS audio-playlist* | *Wilcoxon statistic* | *Z score* |  |
| Average weekly energy burn | 14337.5 | -1.4 | 0.14 |
| Average weekly vigorous activity | 14605 | -1.13 | 0.26 |
| Average weekly moderate activity | 14204 | -1.71 | 0.09 |
| Average weekly light activity | 14417 | -1.34 | 0.18 |
| Average weekly total activity | 14362.5 | -1.44 | 0.15 |
